# Anticancer Drugs: Recent Strategies to Improve Stability Profile, Pharmacokinetic and Pharmacodynamic Properties

**DOI:** 10.3390/molecules27175436

**Published:** 2022-08-25

**Authors:** Giuseppina Ioele, Martina Chieffallo, Maria Antonietta Occhiuzzi, Michele De Luca, Antonio Garofalo, Gaetano Ragno, Fedora Grande

**Affiliations:** Department of Pharmacy, Health and Nutritional Sciences, University of Calabria, 87036 Rende, Italy

**Keywords:** cancer therapy, drug stability, prodrugs, vesicular systems, nanoparticles, trastuzumab

## Abstract

In past decades, anticancer research has led to remarkable results despite many of the approved drugs still being characterized by high systemic toxicity mainly due to the lack of tumor selectivity and present pharmacokinetic drawbacks, including low water solubility, that negatively affect the drug circulation time and bioavailability. The stability studies, performed in mild conditions during their development or under stressing exposure to high temperature, hydrolytic medium or light source, have demonstrated the sensitivity of anticancer drugs to many parameters. For this reason, the formation of degradation products is assessed both in pharmaceutical formulations and in the environment as hospital waste. To date, numerous formulations have been developed for achieving tissue-specific drug targeting and reducing toxic side effects, as well as for improving drug stability. The development of prodrugs represents a promising strategy in targeted cancer therapy for improving the selectivity, efficacy and stability of active compounds. Recent studies show that the incorporation of anticancer drugs into vesicular systems, such as polymeric micelles or cyclodextrins, or the use of nanocarriers containing chemotherapeutics that conjugate to monoclonal antibodies can improve solubility, pharmacokinetics, cellular absorption and stability. In this study, we summarize the latest advances in knowledge regarding the development of effective highly stable anticancer drugs formulated as stable prodrugs or entrapped in nanosystems.

## 1. Introduction

The Global Cancer Incidence, Mortality and Prevalence (GLOBOCAN) is an interactive web-based platform that provides cancer statistics estimating the incidence and mortality for 36 types of cancer and all cancer sites combined in 185 countries. According to data collected in 2020, it has been estimated that one in five people worldwide develop cancer in their lifetime, while one in eight men and one in eleven women die from the disease. The aging population growth, as well as socio-economic risk factors, could contribute to the increase in these estimated numbers [1].

Cancer treatment options include surgery, radiation and chemotherapy, or a combination of them. Chemotherapy is a systemic approach and consists of administering one or more chemicals that can damage fast-growing cells, such as cancerous ones. However, these agents, being non-selective, usually damage healthy cells and tissues with rapid turnover, causing severe toxic effects. The rapid emergence of drug resistance, the instability of the molecules and the poor solubility in water, which makes them unable to permeate through cell membranes, represent further drawbacks of chemotherapy. To overcome these limitations, two or more chemotherapeutics are usually used in combination. Other therapeutic strategies to treat different types of cancer are based on the use of small molecules, including genes, small RNAs and plasmids, which, however, show limitations due to their poor stability in vivo [2].

These disadvantages of conventional anticancer drugs are the reason why the development of alternative treatments with reduced adverse side effects and improved therapeutic efficacy is still demanding. An effective strategy to increase the selectivity of chemotherapeutics involves the use of prodrugs. The latter are inactive compounds that are chemically or enzymatically metabolized in the active drug, reducing the systemic toxicity of conventional therapies [3]. Furthermore, prodrugs can be useful in reducing drug toxicity. For example, although the efficacy of transition metals is widely recognized, due to their intrinsic toxicity, they are generally not included in drug therapies. The design of transition-metal-based prodrugs could, therefore, make them less toxic, allowing the drug to reach therapeutically useful levels [4]. Prodrug therapy, therefore, provides an alternative approach to designing less reactive and less cytotoxic drugs. The design of these new compounds could also help to overcome pharmaceutical, pharmacokinetic and pharmacodynamic hindrances. In fact, they can be used to increase solubility and improve chemical stability and organoleptic characteristics, such as the flavor of the drugs. In particular, they can be designed to improve the absorption throughout the blood–brain barrier or to increase the therapeutic index, as well as the site-of-action selectivity [5]. Since these agents offer a number of advantages, to date, several prodrug formulations have been developed and effectively used for the treatment of different forms of cancer (Table 1). 

The incorporation of anticancer drugs into drug delivery systems (DDS) represents another approach to successfully address pharmacological and pharmacokinetic limitations and to directly carry drugs to the therapeutic site of action while reducing adverse side effects. Accordingly, innovative nanotechnologies had a profound impact on clinical therapeutics, including anticancer drugs [69,70]. Among the most studied incorporation systems, vesicular matrices, such as niosomes, cubosomes or polymeric systems, have shown the best results [71,72,73]. Innovative targeting approaches can also be represented by nanocarriers containing chemotherapeutics conjugated to molecules able to bind to overexpressed antigens (monoclonal antibodies, mAb) [74,75,76,77].

The stability of a drug is verified during all stages of development, through investigations carried out both on the active ingredients and on the final formulation. The analytical methods are generally based on the directives contained in the ICH (International Conference on Harmonization) Guidelines to ensure the safety, efficacy and quality of the drugs tested. In accordance with this document, the stability tests are carried out in different environmental conditions of conservation (pH, temperature, light, air and humidity) [78,79,80]. In the quality control of a drug, the analytical method is carefully selected based on the characteristics of the drug or its formulation to measure the quantity of the drug residual over time and its possible by-products. In general, chromatographic procedures represent the most commonly used technique, both for the separation and quantization of analytes.

Furthermore, the stability of many antineoplastic drugs has been studied in surface waters and wastewater treatment effluents as these compounds, once in the environment, can be harmful to aquatic organisms as they are mutagenic, genotoxic, cytotoxic, carcinogenic and teratogenic [81].

In this work, the most recent findings in this field have been reviewed, focusing in particular on recent strategies effectively used to assess the stability profile of anticancer prodrugs and drugs and to improve their pharmacokinetic and technological profiles. 

Most of the reviews published in the literature focus on the drawbacks of anticancer drugs [82,83] or the use of nanocarriers as DDS [84,85]. In this survey, all the results published to date on the methodologies used to overcome the pharmacokinetic and pharmacodynamic limits of these drugs, as well as to guarantee the improvement in their stability profile, have been collected. In particular, the advantages of using prodrugs and/or incorporating drugs or prodrugs into vesicular systems were thoroughly examined. These approaches favor the therapeutic agent in reaching the site of action at effective concentrations while significantly reducing toxic effects. The benefits and drawbacks of the use of mAb or other experimental strategies for overcoming the limits of conventional drugs have also been discussed.

## 2. Stability of Anticancer Drugs

The stability of most anticancer compounds has been tested under different experimental conditions. Given the alarming concentration of some antineoplastic agents or their degradation compounds found in hospital sewer drains [86,87] or wastewater [88], several studies have focused on evaluating their presence in the environment. In this context, all stability experiments have been conducted by exposing the drugs to mild conditions, such as room temperature and natural pH of the water used as a solvent [89]. For example, a number of cytostatic drugs, including daunorubicin, doxorubicin, vinblastine, chlorambucil, vincristine, irinotecan and melphalan, have been found to be highly unstable in milli-Q water (pH of 6.3) due to the presence of reactive groups in their chemical structures, which favor hydrolytic reactions [90]. In particular, daunorubicin, doxorubicin, irinotecan and vincristine have rapidly degraded, and only 10% of the initial concentration has been detected after 5 min of exposure. On the other hand, vinblastine, chlorambucil and melphalan have been degraded during the first 240 min. The stability has been evaluated in an aqueous environment by varying parameters, such as pH and/or temperature. In water, Mitoxantrone degraded into four stable breakdown products [91], which were identified using liquid chromatography coupled to mass spectrometry (LC–MS). This drug suffered a rapid change in its conformation, resulting in the formation of toxic transformation products that remained unaltered and stable in water for up to two days. Busulfan (1,4-butanediol dimethanesulfonate), an alkylating agent widely used for the treatment of chronic myeloid leukemia, has shown considerable instability in aqueous preparations [92]. The degradation, due to precipitation phenomena, appears to be temperature-dependent: as the storage temperature rises, the stability of the diluted solutions decreases. Busulfan is administered by infusion, but, once prepared in a formulation made from a concentrate, it has a relatively short shelf life. The stability of the solution increases only slightly when stored at 2–8 °C, regardless of the container material used.

Using inductively coupled plasma mass spectrometry (ICP-MS), it was possible to assess the stability profile and the presence in hospital wastewater of cytostatic derivatives of platinum (CPC), antineoplastic agents widely used in clinical applications. These compounds, excreted by treated patients, reach aqueducts and sewers, causing detrimental effects on biota, even at low concentrations [87]. Despite all the compounds belonging to the CPCs class, such as oxaliplatin, carboplatin and cisplatin, having a similar chemical structure, their behavior in the environment is quite different. In fact, these compounds in the environment undergo the processes of hydrolysis, photolysis, dilution, adsorption, sedimentation of suspended solids and biodegradation differently, leading to distinct unaltered compounds or degradation products [93]. Cisplatin products are more easily absorbed on the soil surface compared to carboplatin and oxaliplatin derivatives due to the formation of H-bonds or electrostatic interactions with aqueous soil groups. The stability of carboplatin in aqueous solution is more closely related to nucleophiles concentrations and the pH of the medium, and, in all cases, this activation process is slower than that of cisplatin. Oxaliplatin produces reactive species that contaminate groundwater depending on the composition of the aqueous solution [94].

Several other studies on the stability of anticancer drugs have focused on the evaluation of the degradation profile and the formation of transformation products directly after their exposure to stress conditions, as in the case of imatinib, a highly potent tyrosine kinase inhibitor used as a first-line anticancer drug in the treatment of chronic myeloid leukemia [95]. The photocatalytic degradation kinetics of this compound have been studied under heterogeneous photocatalysis produced in the presence of radicals and the degradation mechanism has been elucidated from LC–MS analysis. In total, 12 transformation products have been detected, and in silico toxicity tests showed that some of these molecules have structural motifs potentially capable of damaging DNA. The stability of 5-fluorouracil, one of the most widely used chemotherapy agents for the treatment of different types of cancer, has been studied under different stressful conditions using high-performance liquid chromatography and infrared spectroscopy. As a result, the drug has shown good stability when exposed to UV radiation, slight degradation at 275 °C and greater degradation at 285 °C, a degradation of about 22% under acid hydrolysis conditions and approximately 97% under alkaline ones and a degradation from 26% to 41% when exposed to oxidative conditions [96].

## 3. Stability of Anticancer Prodrugs

Prodrugs are usually pharmacologically inactive precursors of therapeutic agents, which are chemically or enzymatically transformed within the host into one or more active metabolites. The ability of a prodrug to improve the pharmacokinetic profile or stability of a drug is well known [97]. Different approaches, including the use of vector- or bio-precursor-linked prodrugs, have been developed to ensure that a drug reaches its target in a proper concentration. This approach allows overcoming several drawbacks, including poor water solubility, chemical instability, inadequate oral or local absorption, too short half-life and also formulation or administration issues, facilitating the accumulation of a drug at the desired site of action and thus improving its selectivity and safety [98]. Since a prodrug is transformed into the corresponding active metabolite in vivo, stability studies should be performed on both forms of the drug [81,87,98]. 

As an example, water sorption represents the primary cause of capecitabine degradation. This process is influenced by higher temperature and humidity; in fact, the degradation is accelerated at 40 °C in 75% RH. The application of thermoanalytical techniques and HPLC analyses have proven the stability of capecitabine after 6 months of storage at 25 °C in 60% RH [99]. The degradation behavior of irinotecan hydrochloride has been investigated under different ICH-recommended stress conditions using liquid chromatography–mass spectrometry showing the formation of seven degradation products in pharmaceutical dosage forms. The prodrug has been exposed to oxidative, acid, base, hydrolytic, thermal and photolytic conditions with significant degradation in oxidative, base hydrolysis and photolytic conditions [100]. The stability of floxuridine and leucovorin calcium in combined therapy has been tested at various concentrations and temperature conditions. Both the compounds were stable after 48 h at each tested condition. However, leucovorin calcium underwent degradation, more noticeable at low concentrations, at near-physiologic body temperature compared to other temperatures (4–8 °C and 20 °C) [101].

In combination therapies, the degradation of a drug could be influenced by the chemical characteristics of each component. The physical compatibility and chemical stability of irinotecan, diluted in 5% dextrose in water and combined with the racemic form of leucovorin, have been assessed after the formulation, unprotected from light, has been stored at 23 °C. The solutions remained clear and colorless throughout the 24-h study period for all the tested concentrations of the drugs. On the other hand, in the formulation prepared with a low concentration of irinotecan (0.30 mg/mL) and a high concentration of leucovorin (3.60 mg/mL), a rapid degradation of irinotecan was observed, most likely due to the higher pH of the solution caused by the high concentration of leucovorin [102].

Light degradation of anticancer drugs frequently results in transformation products that are also responsible for toxic effects. The photodegradation of cyclophosphamide and iphosphamide has been investigated using ruthenium-doped titanate nanowires in distilled water and wastewater under UV–vis irradiation. The results indicated that ruthenium exhibited photocatalytic activity for both the drugs, leading to the formation of four photodegradation products for cyclophosphamide and six for isophosphamide. These products have been identified by high resolution mass spectrometry, confirming a higher concentration in wastewater with respect to distilled water. These results have demonstrated that environmental matrices can produce different transformation products and that the experimental conditions in photodegradation studies are critical and should, therefore, be as similar as possible to those of environmental systems [81]. Dacarbazine, an alkylating agent commonly used in combination with other chemotherapeutic agents for the treatment of metastatic malignant melanomas, Hodgkin’s lymphoma and pheochromocytomas, is converted by light into 4-diazoimidazole-5-carboxamide [103]. This photo-transformation product is often responsible for the pain reactions observed during peripheral intravenous infusion during clinical application. The photodegradation profile of the drug solutions was determined using HPLC coupled to UV detection. The study demonstrated that photoproduct production increases in a time-dependent manner up to 4 h at 4 and 25 °C despite the sample being light-shielded, suggesting that light shielding is not required in sample preparation.

## 4. Stability of Anticancer Monoclonal Antibody

Nowadays, significant breakthroughs have been achieved in cancer therapy by applying mAb-based immunotherapy as the antibodies are able to directly target cancerous cells while simultaneously promoting the induction of long-lasting immune responses against cancer cells. However, despite this approach having proven to be very effective for the treatment of different forms of cancer, several drawbacks have yet to be overcome. In particular, drug resistance and poor stability due to the glycoprotein nature of mAb continue to be the major hurdles.

The mechanisms responsible for their instability are either chemical or physical. Several parameters and conditions, including the structure of the proteins, temperature and exposure to light, affect mAb stability [104]. The main process related to chemical degradation is oxidation, which can occur both spontaneously or in the presence of oxidizing agents, such as peroxides or metals. Some amino acid residues, such as methionine and cysteine, are particularly sensitive to oxidation [105]. In addition, asparagine residues can undergo acid-base deamidation, and, as a result, a succinimide intermediate is formed and hydrolyzes spontaneously to aspartic or isoaspartic acid [106].

Variations in temperature or pH can induce the unfolding of proteins, leading to a direct loss of mAb functions and favoring their aggregation, which represents the main cause of physical instability. During protein aggregation, misfolded proteins assemble each other to form high molecular weight species (multimers), such as oligomers and insoluble aggregates, through the formation of non-specific weak bonds, including Van der Waals interactions, hydrogen bonds, hydrophobic and electrostatic interactions, without affecting the primary structure of the molecules [107]. Furthermore, in highly concentrated formulations, due to the increase in viscosity, the formation of aggregates becomes irreversible, leading to problems during the production or the drug administration processes. In general, ingredients such as salts, amino acids, sugars, polyols or surfactants are added to the formulations to overcome these phenomena. In this context, bis-acetyl-lysine and propionyl serine have been identified as more efficient agents compared to the commonly used excipients to minimize the antibody solution viscosity while preventing protein–protein interactions [108].

The presence of several aromatic amino acid residues in the primary structure of mAb makes them particularly sensitive to light, thereby inducing photodegradation with the formation of oxygenated radicals but also fragmentation and cross-linking. The effect of light on mAb aggregation should be investigated in both the original drugs and the final diluted formulations. Despite light not seeming to be involved in a direct alteration of the secondary and tertiary structures of the mAb [104], it has been demonstrated that light exposure promoted the aggregation of monomeric and dimeric fractions of an IgG1 monoclonal antibody. In particular, after the mAb exposure to controlled irradiation, segments with greater flexibility in the C_H_2 and C_H_3 domains of both dimensional fractions and reduced flexibility in some segments of the F_ab_ and C_H_1 domains in the dimer fraction have been identified by mass spectrometry analysis [109].

The effect of light on mAbs aggregation should be investigated on both the original formulation and the diluted preparation adopted in clinical practice. Hernández-Jiménez et al. [110] have performed accelerated photodegradation studies on the commercial drug and on the NaCl commonly diluted formulation of five mAbs (bevacizumab, cetuximab, infliximab, rituximab and trastuzumab). The photodegradation profile has been evaluated by size exclusion chromatography, demonstrating the formation of the aggregates due to the effect of light, in each experiment. This process resulted in mAb fragmentation and consequent aggregation, which were more frequently found in diluted rather than concentrated solutions. Accordingly, the aggregation phenomenon is related to the concentration and nature of mAb both when the formulations are exposed to light and in other stressful conditions, such as freeze/thaw cycles, for all drugs studied. All mAbs underwent degradation with consequent aggregation and/or disruption of the protein chains, probably due to the breakdown of the cystines between the two heavy chains [111]. Despite having a similar IgG1 structure, bevacizumab and rituximab were stable when stored at 4 °C and in freeze/thaw cycles, with a limited aggregate formation, while infliximab and cetuximab degraded even under mild conditions [112,113]. Thanks to the exclusive three-dimensional structure stabilized in the final formulation of Herceptin^®^, trastuzumab resulted as the least light-sensitive antibody despite not being the most concentrated [111]. 

In addition, the use of surfactants in formulations can induce secondary structural changes [114]. The effect of different concentrations of a non-ionic surfactant, sodium dodecyl sulphate, has been investigated in bevacizumab formulations, demonstrating classical aggregate formation only at medium concentrations (0.5–2 mM) of the surfactant. Conversely, at low concentrations (0–0.2 mM), structural changes were observed on both the β sheet and the α helix, producing a disordered structure. At high concentrations of surfactant (3–5 mM), the formation of disordered structures increased.

In conclusion, mAbs are currently one of the most important classes of biotechnological drugs for the treatment of diseases with increasing incidence in the population, such as cancer, autoimmune, inflammatory, infectious and degenerative diseases, and, since the beginning of the COVID-19 pandemic, they have been explored as potential therapeutic tools. Therefore, stability studies are crucial during the development of therapeutic proteins to ensure the quality and safety of the final medicine. Deeper knowledge of the mechanisms involved in a protein can help to avoid the onset of conformational and colloidal changes that reduce its therapeutic efficacy.

## 5. Anticancer Drugs in Nanoparticle Systems

The development and application of vesicular systems capable of ensuring controlled delivery of anticancer drugs to the desired site of therapeutic action in adequate quantities to exert their actions are increasing. These systems improve therapeutic efficacy while reducing negative side effects, providing many advantages, including improved pharmacodynamic and pharmacokinetic profiles, which result in a prolonged half-life and enhanced drug stability, ensuring protection from chemical or physical degradation [115,116]. Since most antineoplastic agents are very sensitive to different conditions, in clinical practice, improvement in the drug stability profile can simplify the work of pharmacists during the preparation of different formulations, and of healthcare professionals when handling the drugs that need to be administered in hospital care [117]. Furthermore, improving the stability of anticancer agents could facilitate home therapy as the drugs could be supplied to patients via portable elastomeric pumps without risking their alteration and, therefore, treatment failure. 

The currently available nanocarriers for anticancer drugs vary in structures, sizes and physicochemical properties. These systems can be of natural origin, and, therefore, made up of simple structures derived from phospholipids, such as lecithin, and of synthetic nature and thus characterized by more complex structures consisting of polymers sometimes complexed with metals. Niosomes (non-ionic surfactant vesicles) are one of the most commonly applied carriers for anticancer drugs. These vesicles are obtained by the hydration procedure of a non-ionic surfactant with cholesterol in which the surfactants form a closed bilayer vesicle in an aqueous medium based on its amphiphilic nature. In this structure, the surfactant molecules are oriented away from the solvent so that the hydrophilic ends of the non-ionic surfactant point outwards and the hydrophobic ends face each other to form the bilayer, whereas the hydrophilic heads remain in contact with the aqueous solvent. As for the natural liposomes, the properties of the niosomes depend on the composition of the vesicles, size, lamellarity, tapped volume, surface charge and concentration. However, unlike niosomes, liposomes are expensive, and their components, such as phospholipids, easily suffer oxidative degradation. This behavior requires special storage conditions and makes liposomes challenging to handle [84]. All these structures include both aqueous compartments for the incorporation of hydrophilic molecules and lipid layers for the transport of lipophilic molecules [116]. 

Over the last few decades, the use of nanoparticle (NP)-based DDS has shown numerous advantages in cancer treatment, including the ability to overcome drug resistance caused by overexpression of drug efflux transporters, defective apoptotic pathways and a hypoxic environment [85]. For example, NPs can avoid the exposure of anticancer drugs to efflux transporters as they enter the cell primarily through endocytosis rather than diffusion. Usually, the type of NPs used in cancer therapy (organic, inorganic or hybrid) is designed or chosen based on their size and characteristics, as well as the pathophysiology of the tumors. Organic NPs include liposome- and polymer-based NPs, such as micelles and dendrimers, whereas inorganic NPs include gold NPs (Au-NPs), carbon nanotubes, silica NPs, magnetic NPs and quantum dots; finally, the hybrid NPs that combine the advantages of the different types include the lipid–polymer, organic–inorganic hybrid NPs and cell-membrane-coated NPs.

**Figure 1:**
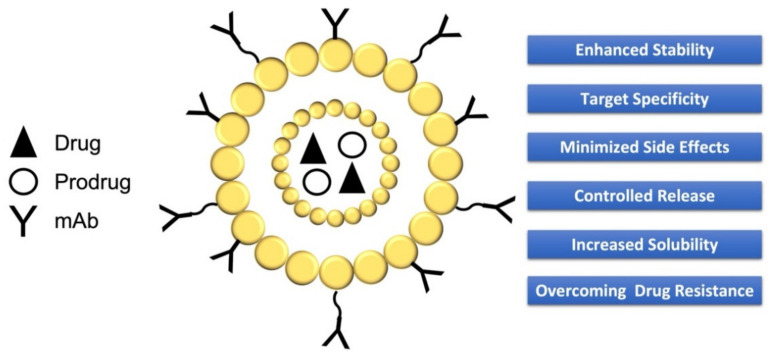
NP entrapping drugs and prodrugs coated with mAbs. List of advantages in the use of NPs.

Figure 1 depicts an NP entrapping a drug or prodrug coated with mAb and the advantages in the use of this system.

Table 2 lists most of the applied inclusion systems for anticancer drugs and prodrugs and the advantages obtained from the proposed formulation.

To date, several studies dealing with the incorporation of anticancer drugs into supramolecular systems have been published in the literature, and, in all cases, an improvement in the chemical–physical stability of the drug, and, consequently, better therapeutic efficacy, have been observed. Some examples are given below. Paclitaxel targeting has been improved by its inclusion in natural milk-derived exosomes. This compound is known to have poor solubility in water, while the formulation in exosomes can ensure a continuous release up to 48 h with an ideal stability profile for clinical applications [157]. Higher thermal stability of methotrexate has been obtained by encapsulation in novel targeted systems. Dhanka et al. have proposed the loading of the drug into gellan gum microparticles prepared by using a simple water-in-oil emulsion solvent diffusion method [124]. Improvement in thermodynamic stability has also been obtained by Mishra et al., who incorporated methotrexate into novel-targeted Pluronic (PEOPPO- PEO tri-block co-polymer) F127 polymeric micelles proposed for intravenous administration in MCF7 cancer cells [125]. Polymeric NPs prepared starting from N-(2-hydroxypropyl)methacrylamide have been used to entrap bortezomib, improving its stability and bioavailability [189]. The efficacy of nanostructured lipid carriers containing imatinib has been tested in vitro in MCF-7 breast cancer cells. In this case, vesicles have been prepared using fat and oil by the hot homogenization method, and sodium lauryl sulphate (SLS) and T80 have been used as surfactants for the stabilization of the system [193]. Due to their small size (~ 100 nm) and lipid nature, these particles may ensure adequate drug penetration through membranous barriers, leading to a significant improvement in the therapeutic efficacy.

The effect of temperature on the stability of lipid nanocarriers has also been verified. As a result, temperature affected several parameters of the prepared formulations, including particle size, polydispersion index, encapsulation efficiency and zeta potential, after a three-month storage period. In particular, an increase in the size of the particles has been observed, probably due to the swelling or adsorption of surfactants on their surfaces, which, however, remained in the colloidal nanometer range (<550 nm), confirming the absence of aggregation.

### 5.1. Anticancer Prodrugs in Nanoparticles Systems

As described in Section 3, despite the promising anticancer potential of many anticancer prodrugs, their clinical use is limited due to sensitivity to acid and enzymatic hydrolysis. To overcome these limitations, prodrugs have also been incorporated into different controlled delivery systems. As an example, capecitabine has been formulated in co-polymeric hydrogel as a smart pH-responsive network to facilitate its oral administration, reducing its sensitivity to gastric pH [118]. To overcome some of the therapeutic disadvantages of 6-thioguanine, a supramolecular ternary system, involving the inclusion of the drug in β-cyclodextrins (βCD) and a subsequent interaction of the βCD-thioguanine complex with gold NPs, has been proposed. This strategy promoted increased solubility and improved the stability of the incorporated prodrug, ensuring, among other advantages, site-specific transport due to their nanometer size [126]. Chitosan-based polyelectrolyte complexes, based on orientated superparamagnetic NPs, have been developed to perform targeted delivery of irinotecan at the tumor site under the effects of a magnetic field. These complexes were prepared starting from chitosan and polyglutamate via an all-in-water process, thereby excluding the use of any potentially toxic chemicals while reaching higher stability and, consequently, better efficacy of the inclusion complex compared to the free drug against colon cancer cells [168].

### 5.2. Combination Therapy in Nanoparticles Systems

Nowadays, combination therapy is a widely adopted strategy for cancer treatment since acting simultaneously on multiple targets allows the reduction in the dose for each single drug and slows down the onset of drug resistance. Recently, vesicular systems for encapsulating combination drugs have been designed to further improve efficacy. Fludarabine/mitoxantrone combination therapy has been successfully adopted for the treatment of different types of lymphoma and chronic leukemia. The efficacy of this combined therapy has been further enhanced by co-incapsulating both compounds in liposomes: fludarabine has been passively encapsulated during liposome formation, while the loading of mitoxantrone has been driven by a transmembrane pH gradient. This formulation would not only represent a promising and efficient therapeutic strategy but could also improve the long-term stability of both drugs, as evidenced by a recent study after a three-month monitoring period. [106].

Liposome encapsulating polymeric micelles loaded with vinorelbine and cis-diamminedichloroplatinum (II) (cisplatin) have also been designed for the treatment of non-small-cell lung cancer, an aggressive tumor with high mortality and poor prognosis [166]. The stability of this formulation has been tested in PBS (pH 7.4) solvent and 10% plasma, showing no significant change in particle size and a slight increase in the polydispersity index, indicating that the particles could accumulate if stored for more than 72 h, and, therefore, that the co-delivered drugs were protected from metabolism and rapid elimination.

### 5.3. Monoclonal Antibody in Nanoparticles Systems

Despite their proven efficacy as anticancer drugs, the clinical use of mAbs is severely limited by their poor chemical and enzymatic stability and consequent aggregate formation. A valid strategy to overcome these hurdles and achieve an adequate intracellular release of non-aggregated antibodies in the desired site of action consists of the encapsulation of the mAb into polymeric or lipid NPs. Because these systems are resistant to several chemical and physical factors, including body temperature, they can protect the antibody during the drug’s persistence in the bloodstream. Furthermore, when the NPs are endocytosed by the tumor cells, they release the antibody molecules inside the cytoplasmic compartment, avoiding the action of lysosomes and thus preventing enzymatic degradation. Bevacizumab-loaded NPs performed well as a controlled release system, also slowing down enzymatic degradation [176,178,179]. Bevacizumab lipid NPs have been developed as an innovative delivery system for intravitreal injection capable of ensuring high drug stability [177]. Furthermore, such a formulation improved the drug intraocular bioavailability and patient compliance by avoiding repeated intravitreal injections. The addition of choline dihydrogen phosphate, a promising biocompatible ionic liquid for mAb formulation, resulted in a significant improvement in therapeutic efficacy due to the suppression of unfolding and aggregation of trastuzumab, justifying its use for the preparation of stable therapeutic antibody formulations [186].

The association of docetaxel with trastuzumab is a therapeutic regimen successfully used to treat breast cancer. Docetaxel, commonly dissolved in Tween 80 surfactant for its clinical formulations, frequently causes severe hypersensitivity and other adverse reactions. The development of NPs loaded with both drugs can be useful to overcome the single drug drawbacks while improving the therapeutic efficacy of the combined treatment. Lipid–polymer hybrid NPs have been prepared for this purpose by combining poly(D,L-lactide-co-glycolide), polyethylenimine and lipids to form a hydrophobic core. Trastuzumab has been electrostatically adsorbed on the surface of these NPs as a ligand targeting human epidermal growth factor receptor 2 (HER2)-positive breast cancer cells, while Docetaxel is entrapped in core NPs. The stability of the proposed formulation has been studied under physiological (37 °C) and storage conditions (4 °C), and the effect of the dilution has been tested at a concentration of 1.0 mg/mL with PBS (0.02 mol/L, pH 7.4) and PBS with 10% fetal bovine serum (vol/vol), showing very good stability during storage, transportation and use [184]. The efficacy of the same combination of drugs has been tested by preparing stealth liposomal docetaxel with engrafted trastuzumab on its surface [185]. Two formulations of liposomes with several engraftment techniques have been tested: a neutral formulation using phosphatidylcholine (antibody nanoconjugate-1) or a positive formulation using 1,2-dioleoyl-3-trimethylammonium-propane. Stability studies confirmed a very good performance at 4 °C or 25 °C as a light-protecting system for up to 1 week.

## 6. Conclusions

Despite their substantial contributions to cancer treatment, all conventional chemotherapy drugs suffer from several drawbacks, including rapid elimination, poor bioavailability, low intratumoral release, non-specific cytotoxicity and consequent systemic side effects, which are frequently followed by the onset of drug resistance. Over the past decade, to overcome these limitations, a large number of drug delivery systems have been developed, resulting in a significant improvement in the pharmacodynamic and pharmacokinetic profiles of the drugs, as well as in their physicochemical stability. Polymeric or lipid nanoparticles represent the most commonly used systems for incorporating anticancer drugs and preventing aggregation in monoclonal antibody formulation. Several prodrugs are incorporated into cyclodextrin matrices, which are well known for their ability to improve the solubility profile of the incorporated compounds.

The therapeutic efficacy of anticancer agents included in nanosystems has now been widely established since they ensure a controlled release of an adequate amount of the drug at the desired site of action and reduce the drug sensitivity to physicochemical factors during the preparation, managing and storage phases. The possibility of including in the same vehicle two or more drugs in combination offers further advantages by allowing the reduction in the dosage of each drug and, therefore, the toxicity. In these cases, larger vesicles, such as liposomes, are used. Several studies focusing on the development of innovative formulations are still ongoing. Such systems, some of which have already been approved, and many others that are in clinical or preclinical development stages, offer great hope for safer and more efficient options to be adopted in the near future for cancer treatment.

## Figures and Tables

**Table 1:** List of the anticancer drugs and prodrugs and the diseases in which they are most used.

Drug Classes	Active Compound	Prodrug	Diseases	Ref.
Antimetabolites	Mercaptopurine	Azathioprine	Acute lymphoblastic leukemia	[6,7]
	5-Fluorouracil	Capecitabine	Breast cancer, esophageal cancer, laryngeal cancer, gastrointestinal and genitourinary tract cancer	[8]
	Deoxyadenosine	Cladribine	Hairy cell leukemia	[9,10]
	1-β-D-arabinofuranoside 5′-triphosphate	Cytarabine	Acute myeloid leukemia	[11]
	9-beta-D-arabinosyl-2-fluoroadenine	Fludarabine	Chronic lymphocytic leukemia	[11]
	5-Fluorouracil		Different types of neoplasms	[8]
	Gemcitabine diphosphate and triphosphate	Gemcitabine	Solid cancers	[11]
	6-Mercaptopurine		Acute lymphoblastic leukemia	[7,11,12]
	Methotrexate		Several kinds of cancer, such as colon cancer	[13]
	6-Thioguanosine	6-Thioguanine	leukemias, lymphomas, mesothelioma, melanoma, biliary tract cancer, glioblastoma, osteosarcoma, soft tissue sarcoma, neuroendocrine tumors and lung, pancreatic and squamous cell carcinomas	[14,15]
	5-Fluorouracil	Floxuridine	Liver cancer	[6,16]
	Methyl-tetrahydrofolate	Leucovorin	Acute lymphoblastic leukemia	[17,18]
Alkylating agents	Busulfan		Chronic myelogenous leukemia	[19]
	Carmustine		Glioblastoma multiforme	[20]
	Acrolein and phosphoramide mustard	Cyclophosphamide	Several kinds of cancer and autoimmune disorders	[21,22]
	5-aminoimidazole-4-carboxamide	Dacarbazine	Malignant melanoma or sarcoma	[23]
	Lomustine		Brain tumors	[24]
	Mechlorethamine		Mycosis fungoides	[25]
	Melphalan		Multiple myeloma	[26]
	Azo-Procarbazine	Procarbazine	Hodgkin’s lymphoma	[27,28]
	Triethylenethio-phosphoramide	Thiotepa	Ovarian cancer, breast cancer and superficial bladder cancer	[29,30]
	Semustine		Lewis lung carcinoma, leukemia, metastatic brain tumor, Hodgkin’s lymphoma, malignant melanoma and lung carcinoma	[31]
Anthracyclines	Daunorubicin		Leukemia	[32]
	Doxorubicin		Leukemia, breast cancer	[32]
	Epirubicin		Breast cancer	[33]
	Idarubicin		Acute leukemia	[34]
	Mitoxantrone		Breast and prostate cancers, lymphomas and leukemias	[35]
Antitumor antibiotic	Bleomycin		Hodgkin’s and non-Hodgkin’s lymphoma, renal, cervical, laryngeal, testicular, lung and others	[36]
	Dactinomicyn		Different solid cancer	[37]
	Mitomycin		Adenocarcinoma of the stomach	[38]
	Plicamycin		Testicular and germ cancers	[39]
Epipodophyllotoxins	Etoposide		Small-cell lung cancer, leukemia, lymphoma, breast and ovarian carcinomas, testicular cancer	[40]
	Teniposide		Small-cell lung cancer, leukemia	[41]
Taxanes	Cabazitaxel		Prostatic cancer	[42]
	Docetaxel		Metastatic prostate cancer	[43]
	Paclitaxel		Ovarian, breast and lung cancer, as well as Kaposi’s sarcoma	[44]
Vinca alkaloids	Vinblastine	Vinblastine-N-Oxide	Pancreatic ductal adenocarcinoma	[45]
	Vincristine		Precursor B-cell acute lymphoblastic leukemia	[46]
	Vinorelbine		Non-small-cell lung cancer and metastatic breast cancer	[47]
Campotothecins	SN-38 (7-ethyl-10-hydroxy-camptothecin)	Irinotecan	Solid tumors, including colorectal, pancreatic and lung cancer	[48]
	Topotecan		Cervical cancer	[49]
Platinum analogs	Carboplatin		Ovarian cancer cells	[50]
	Cisplatin		Solid cancers, such as testicular, ovarian, head and neck, bladder, lung, cervical cancer, melanoma, lymphomas and several others	[50,51]
	Oxaliplatin		Colorectal cancer	[52]
Monoclonal antibody	Bevacizumab		Metastatic colorectal cancer, metastatic breast cancer, non-small-cell lung cancer, glioblastoma, renal-cell carcinoma, ovarian cancer and cervical cancer	[53]
	Cetuximab		Non-small-cell lung cancer	[54]
	Rituximab		Lymphoid malignancies, including aggressive forms of B-cell non-Hodgkin lymphoma, B-cell malignancies, follicular lymphoma, diffuse large B-cell lymphoma, chronic lymphocytic leukemia and mantle cell lymphoma	[55]
	Trastuzumab		Breast and metastatic gastric cancer	[56]
Growth inhibitor	Axitinib		Renal-cell carcinoma	[57]
	Bortezomib		Multiple myeloma	[58]
	Bosutinib		Philadelphia chromosome-positive chronic myelogenous leukemia	[6]
	Crizotinib		Non-small-cell lung cancer	[59]
	Dabrafenib		BRAF-mutated melanoma	[60]
	Dasatinib		Chronic myeloid leukemia and Philadelphia chromosome-positive acute lymphoblastic leukemia	[61]
	Imatinib		Chronic myeloid leukemia (CML)	[62]
	Lapatinib		Breast and gastrointestinal cancer	[63]
	Nilotinib		Chronic myeloid leukemia (CML)	[64]
	Pazopanib		Metastatic renal-cell carcinoma	[65]
	Sorafenib		Hepatocellular carcinoma	[66]
	Sunitinib		Renal-cell carcinoma	[57]
	Trametinib		BRAF-mutated melanoma	[60]
	Vandetanib		Metastatic medullary tyroid cancer	[67]
	Vemurafenib		BRAF-mutated melanoma	[68]

**Table 2:** Inclusion systems and their advantages in protecting the anticancer drugs.

Drug	Inclusion Systems	Advantages	Ref.
Capecitabine	Smart pH-responsive co-polymeric hydrogels	Protection from chemical and enzymatic hydrolysis and improvement in the stability in the gastric media	[118]
Cladribine	Nanostabilized polyacrylamide matrix	Better operational stability and mechanical properties	[119]
Cytarabine	Liposomal formulation in hydrogel system	Improvement in stability	[74]
Fludarabina	Co-encapsulation with mitoxantrone in liposomes	Improvement in long-term stability	[120]
5-Fluorouracil	Co-encapsulation with leucovorin in NPs	Improvement in long-term stability	[75,121]
Gemcitabine	Temperature-sensitive liposomes	Improvement in long-term stability	[76]
6-Mercaptopurine	NPs	Improvement in thermal stability	[122]
	Gold NPs	Improvement in stability in diluted aqueous solutions	[77]
	Magnetite NPs	Improvement in thermal stability	[123]
Methotrexate	Gellan gum microparticles	Higher thermal stability	[124]
	Amphiphilic PEO–PPO–PEO tri-block co-polymeric nanomicelles	Improvement in thermodynamic stability	[125]
6-Thioguanine	Inclusion in βcyclodextrin and subsequent interaction with gold NPs	Increase in solubility and improvement in stability	[126]
Floxuridine	Boron nitride nanotube encapsulation	Improvement in long-term stability	[127]
Leucovorin	Co-encapsulation in NPs with of 5-fluorouracil	Improvement in long-term stability	[121]
Busulfan	Encapsulation within water-soluble pillae[5]arene	Reduction in hydrolytic degradation	[128]
Carmustine	Adsorption on the surface of the γ-Fe_2_O_3_ NPs	Improvement in long-term stability	[129]
	Cationic core-shell NPs	Improvement in long-term stability	[130]
Lomustine	Thermosensitive liposomes	Improvement in long-term stability	[131]
Mechlorethamine	Addition of free radical inhibitor for topical use	Improvement in long-term stability	[132]
Melphalan	Liposomal formulation based on a fluid lipid bilayer of natural phospholipids in the form of dioleoylglyceride ester	Improvement in stability in human serum	[133]
Daunorubicin	Liposomes	Improvement in long-term stability	[134,135]
Doxorubicin	Poly(lactide-co-glycolide) NPs with poloxamer 188	Improvement in long-term stability	[136]
	Peptide-based hydrogels and nanogels	Improvement in long-term stability	[137]
	Chitosan-coated nanodiamonds	Improvement in long-term stability	[138]
	PEGylated liposomal nanodrugs	Improvement in long-term stability	[139]
Epirubicin	Drug-eluting beads	Improvement in long-term stability	[140]
	Bifunctional drug-loaded micelles	Improvement in long-term stability	[141]
Idarubicin	Drug-eluting beads	Improvement in long-term stability	[142]
	Drug-eluting embolics beads	Improvement in long-term stability	[143]
Mitoxantrone	Estrone-targeted liposomes	Improvement in long-term stability	[144]
	Hyaluronan magnetic NPs	Improvement in long-term stability	[145]
	Liposomes in PLGA NPs	Improvement in long-term stability	[146]
Bleomycin	Biodegradable chitosan nanogel	Improvement in thermal stability	[147]
Mitomycin	PEGylated liposomes	Improvement in long-term stability	[148]
Etoposide	PLGA NPs	Improvement in long-term stability	[149]
	Nanostructured lipid carriers	Improvement in long-term stability	[150]
Teniposide	Aqueous mixtures of detergent-phospholipid	Improvement in long-term stability	[151]
	Nanosuspensions	Improvement in long-term stability	[152]
Docetaxel	Nanocrystal-loaded micelles	Enhancement in blood circulation	[153]
	Chondroitin sulphate-hybridized zein NPs	Improvement in long-term stability	[154]
Cabazitaxel	Surfactant-stripped micelles	Improvement in long-term stability	[155]
	Albumin NPs	Improvement in long-term stability	[156]
Paclitaxel	Natural exosome	Improvement in stability profile	[157]
	Polymeric micellar system	Increased solubility, greater stability	[158]
	Merocyanine conjugates	Favorable biological stability	[159]
	17-fluorinated ethanol-modified drug in NPs	Robust colloidal stability	[160]
Vinblastine	PEGylated niosomes	Increased solubility in water, reduction in side effects	[161]
Vincristine	Artificial low-density lipoproteins	Improvement in diffusion capacity in tumor tissue and lower toxicity	[162]
	Liposomes	Improvement in efficacy stability	[163]
Vinorelbine	Liposomes prepared with ammonium salts of several anionic agents	Improvement in efficacy and stability	[164]
	Nanomicelles	Reduction in side effects and increase in drug efficacy	[165]
	Liposome encapsulating polymeric micelles. Co-encapsulation with cis-diamminedichloroplatinum (II)	Reduction in toxicity and increase in plasma half-life	[166]
	Intravenous lipid emulsion	Improvement in lipophilicity, and fewer toxic effects	[167]
Irinotecan	Superparamagnetic chitosan nanocomplex	Improvement in effectiveness and biodistribution	[168]
Topotecan	Thiolated chitosan NPs	Improvement in stability and increase in absorption	[169]
	Lipid NPs	Protection from hydrolysis	[170]
Cisplatin	Liposome encapsulating polymeric micelles. Co-encapsulation with vinorelbine	Reduction in toxicity and increase in plasma half-life	[166]
	NPs	Improvement in stability	[171]
Carboplatin	Niosomal nanoplatform	Improvement in stability	[172]
	Conjugation with an arginine-rich triple-helical peptide	Improvement in pharmacokinetic profile	[173]
	NPs	Outstanding plasma stability	[174]
Oxaliplatin	Conjugation with PEGylated-nanobody	Prolonged circulation in vivo	[175]
Bevacizumab	Excipient in dilute solutions	Stabilization in unfavorable conditions, such as low concentration or body temperature. Prevention of aggregation.	[176,177]
	Lipid NPs	Biochemical and biophysical stabilization. Prevention of aggregation.	[178]
	Nanoincapsulation into PLGA NPs	Improvement in long-term stability. Prevention of aggregation.	[179]
Cetuximab	Silica NPs	Improvement in stability and bioavailability. Prevention of aggregation.	[180]
	Chitosan NPs with and without drug conjugation	Improvement in stability and bioavailability. Prevention of aggregation.	[181]
	Polymersome–mertansine nanodrug	Improvement in stability and bioavailability. Prevention of aggregation.	[182]
Rituximab	Iron oxide NPs	Colloidal stability in buffer solution. Prevention of aggregation.	[183]
Trastuzumab	Coated NPs with docetaxel	Prevention of aggregation and improvement in stability and pharmacokinetics profile	[184]
	Stealth immunoliposome coated with docetaxel	Prevention of aggregation and improvement in stability and pharmacokinetics profile	[185]
	Choline ionic liquid vesicles	Prevention of aggregation and improvement in stability and pharmacokinetics profile	[186]
	Drug conjugated with SCN-Bn-NOTA and radiolabeled with ^64^Cu	Prevention of aggregation and improvement in stability and pharmacokinetics profile	[187]
Axitinib	Nanofibrous membranes prepared with poly(ε-caprolactone)/collagen	Improvement in long-term stability	[188]
Bortezomib	Polymeric NPs	Improvement in water solubility chemical stability	[189]
Crizotinib	Thermosensitive liposome	Improvement in targeting efficacy	[190]
Dasatinib	Biodegradable NPs	Improvement in long-term stability	[191]
	H-sensitive targeted micelle system. Co-encapsulation with curcumin	Improvement in long-term stability	[192]
Imatinib	Nanostructured lipid carriers	Improvement in long-term stability at 25 °C	[193]
	Nanocrystal delivery system	Improvement in long-term stability	[194]
Lapatinib	Nanocrystals stabilized with a PEG coating	Improvement in stability for at least 4 days in plasma-containing buffers	[195]
	Polymeric micelles	Improvement in stability	[196]
	Human serum albumin NPs	Improvement in stability	[197,198]
	Incorporation in lipoprotein-like NPs	Improvement in solubility in water and organic solvents	[199]
Sorafenib	Solid lipid NPs	Increase in homogeneity and improvement in physical stability	[200]
	Nucleoside-lipid-based nanocarriers	Increase in homogeneity and improvement in physical stability	[201]
Sunitinib	Self-nanoemulsifying system	Improvement in long-term stability	[202]
	Paclitaxel-loaded micelles	Improvement in long-term stability	[203]
	Self-nanoemulsifying system	Improvement in long-term stability	[204]
Vandetanib	Nanocarrier based on apoferritin	Improvement in drug delivery	[205]
Vemurafenib	Peptide-modified loaded liposomes	Improvement in long-term stability	[206]
